# Four Prizes

**Published:** 1869-12

**Authors:** 


### Four Prizes.

The Editors of the *American Journal of Obstetrics and Diseases of Women and Children* offer the following prizes for the best essays on the subjoined subjects:

1. Fifty dollars (in gold) for the best essay on "Catarrh of the Uterus, its Etiology and Treatment."

2. One hundred dollars (in gold) for the best essay on "The Morbid Anatomy of the Placenta."

3. Fifty dollars (in gold) for the best essay on "Electricity in the Treatment of the Diseases of Infants and Children."

4. One hundred dollars (in gold) for the best essay on "Congenital Deformities and Diseases Depending on Maladies of the Uterus or Membranes."

